# Risk factors and prediction model for mental health in Chinese soldiers

**DOI:** 10.3389/fpsyt.2023.1125411

**Published:** 2023-05-05

**Authors:** Mengxue Zhao, Ying He, Quan Tang, Ni Wang, Haoxin Zheng, Zhengzhi Feng

**Affiliations:** ^1^Department of Military Psychology, Faculty of Medical Psychology, Army Medical University (Third Military Medical University), Chongqing, China; ^2^Department of Psychiatry, Second Affiliated Hospital, Army Medical University (Third Military Medical University), Chongqing, China; ^3^Medical Psychology Department, No. 984 Hospital of PLA, Beijing, China; ^4^General Hospital of Xinjiang Military Region, Wulumuqi, China; ^5^The 33rd Company of the 11th Battalion, College of Command and Control Engineering, Army Engineering University of PLA, Nanjing, China; ^6^Faculty of Medical Psychology, Army Medical University (Third Military Medical University), Chongqing, China

**Keywords:** mental health, risk factors, prediction model, soldiers, Chinese

## Abstract

**Introduction:**

This study aimed to explore potential risk factors for mental health concerns, and the prediction model for mental health concerns in Chinese soldiers was constructed through combined eligible risk factors.

**Methods:**

This cross-sectional study was performed on soldiers under direct command from Gansu, Sichuan, and Chongqing in China, and the soldiers were selected by cluster convenient sampling from 16 October 2018 to 10 December 2018. The Symptom Checklist-90 (SCL-90) and three questionnaires (Military Mental Health Status Questionnaire, Military Mental Health Ability Questionnaire, and Mental Quality Questionnaire for Army Men) were administered, including demographics, military careers, and 18 factors.

**Results:**

Of 1,430 Chinese soldiers, 162 soldiers presented mental disorders, with a prevalence of 11.33%. A total of five risk factors were identified, including serving place (Sichuan vs. Gansu: OR, 1.846, 95% CI: 1.028–3.315, *P* = 0.038; Chongqing vs. Gansu: OR, 3.129, 95% CI, 1.669–5.869, *P* = 0.003), psychosis (OR, 1.491, 95% CI, 1.152–1.928, *P* = 0.002), depression (OR, 1.482, 95% CI, 1.349–1.629, *P* < 0.001), sleep problems (OR, 1.235, 95% CI, 1.162–1.311, *P* < 0.001), and frustration (OR, 1.050, 95% CI, 1.015–1.087, *P* = 0.005). The area under the ROC curve by combining these factors was 0.930 (95% CI: 0.907–0.952) for predicting mental disorders in Chinese soldiers.

**Conclusion:**

The findings of this study demonstrate that mental disorders and onset in Chinese soldiers can be predicted on the basis of these three questionnaires, and the predictive value of the combined model was high.

## Introduction

Mental health concerns are the most prevalent health problems among the adult population, comprising intellectual, spiritual, and emotional health (1). The World Psychiatric Association addressed four mental health standards, including physical and mental coordination, adaptation to the social environment, wellbeing, and use of one's full range of abilities at work (2). Seven mental disorders contribute to the top 25 causes of disability worldwide, including major depressive disorder ranked #2 and anxiety disorder ranked #9 (3). Studies have reported that the prevalence of mental disorders ranged from 9.6 to 27.8% in the adult population across countries (4, 5), while the prevalence of depressive symptoms in Chinese soldiers was 25.20% (6). The deleterious consequences of these illnesses affect quality of life. Therefore, intervention and prevention strategies should be explored based on the risk factors for the early onset of mental disorders, reducing adverse outcomes related to these illnesses.

Risk factors for the onset of mental disorders have already been identified, including excessive pressure or harsh working environments, and most studies focus on the general population (7–11). Several studies have identified risk factors for mental disorders in military personnel and veterans across countries, and the risk factors in German soldiers included alcohol-use disorders and anxiety disorders (12); the risk factor in American active-duty Navy and Marine Corps soldiers was the gender of woman (13); the risk factors in Chinese military personnel included childhood foster care, the gender of woman, stressful life events, younger age, and being divorced/widowed (14); and the risk factor in American service members deployed to Iraq and Afghanistan was FKBP5 (15). Several studies have illustrated the risk factors for mental disorders among Chinese soldiers, and they point out that age, gender, education, residence, military service, social support, and headache, abdomen ache, diarrhea, or training injury during the training course could affect the progression of mental disorders (6, 16, 17). Soldiers face substantial challenges due to their rapid transition from civilian status to training for combat (18). Moreover, military tasks typically present high intensity. Soldiers must train under extreme and harsh conditions, including cold and heat stress and high altitude (19–21). Furthermore, disturbed routines and latent danger can induce intense pressure on soldiers, damaging sleep patterns and promoting subsequent mental disorders (22). It is essential to construct a prediction model for depression in Chinese soldiers, as it has not been developed for clinical practice. The current study was performed to construct a prediction model to identify soldiers at high risk for mental disorders.

## Materials and methods

### Subjects

This study design was cross-sectional, and cluster convenience sampling was applied. A sample of 1,430 Chinese soldiers was recruited by direct command from Gansu, Sichuan, and Chongqing. The Medical Ethics Committee of the Third Military Medical University approved this study. All soldiers provided informed consent to participate after being informed of the purpose of the study. Our study was carried out following the Declaration of Helsinki. The recruited individuals met the following criteria: (1) residence in Gansu, Sichuan, and Chongqing; (2) duration of military service ≥ 1.0 years; and (3) educational level of junior high school or above. Individuals who presented severe physical illness, schizophrenia, bipolar disorder, cerebral organic diseases, epilepsy, or substance abuse were excluded. Missing information for demographics and the military careers of soldiers in the questionnaire and individuals who did not complete the study caused by withdrawal, deployment, injuries, or other causes were excluded.

### Collection indicators

We collected information on demographics and the military careers of all soldiers, including the location of service, gender, age, professional age, and official rank. In addition, 18 factors were collected using three questionnaires, including the Military Mental Health Status Questionnaire (MMHSQ), Military Mental Health Ability Questionnaire (MMHAQ), and Mental Quality Questionnaire for Army Men (MQQAM) (23–25). There were seven (psychosis, depression, suicidal tendency, post-traumatic stress, sleep problems, social phobia, and antisocial tendency), six (emergency-dealing ability, stress tolerance ability, calm ability, adapting ability, physical control ability, and cooperation ability), and five (intelligence, loyalty, courage, confidence, and frustration) items included in MMHSQ, MMHAQ, and MQQAM, respectively. The content, structure, and criterion validity for MMHSQ, MMHAQ, and MQQAM are relatively good (23–25). The detailed items for MMHSQ, MMHAQ, and MQQAM are shown in the Supplementary material.

### Outcome

The mental disorders in the soldiers were assessed using the Symptom Checklist-90 (SCL-90), which includes 90 items. Each item was scored using a 5-point Likert scale. The scores for the SCL-90 range from 90 to 450, reflecting the severity of the symptoms. A total of 10 subscales were included: somatization, obsessive-compulsive, interpersonal sensitivity, depression, anxiety, hostility, phobic anxiety, paranoid ideation, psychoticism, and additional items (26). Mental disorders were defined using the scores on each subscale ≥ 2.0.

### Statistical analysis

All collected data were classified as either categorical or continuous variables. Next, the data for continuous variables were presented as means (standard deviation) or medians (quartile) according to data distribution, while frequencies (proportions) were applied to describe categorical data. The differences in the characteristics of soldiers with and without mental disorders were assessed using *t*-tests, Kruskal–Wallis tests, chi-square tests, and Cochran–Mantel–Haenszel tests according to the data type and distribution. The distribution of mental disorders was assessed using the SCL-90 according to the serving place, and they were also described as frequencies (proportion). After this, a univariate logistic regression was used to explore potential risk factors (serving place, gender, age, duration of military service, official rank, psychosis, depression, suicidal tendency, post-traumatic stress, sleep problems, social phobia, antisocial tendency, emergency-dealing ability, stress tolerance ability, calm ability, adapting ability, physical control ability, cooperation ability, intelligence, loyalty, courage, confidence, and frustration) and subjected to the multivariate logistic regression model, using α = 0.05 and β = 0.10. Next, the receiver operating characteristic (ROC) curve was constructed based on the identified risk factors, and the area under the curve (AUC) was analyzed. All reported *P*-values were two-sided. A *P* < 0.05 was considered statistically significant. All the statistical analyses were conducted using IBM SPSS Statistics for Windows, version 26.0 (SPSS 26.0).

## Results

### Baseline characteristics

Table 1 shows baseline demographics, military careers, and 18 factors from three questionnaires between normal and mental disorder status. Of 1,430 Chinese soldiers, 162 soldiers (11.33%) presented with mental disorders. We found no significant difference in age between soldiers with and without mental disorders. Significant differences were found between the groups for location, gender, duration of military service, official rank, psychosis, depression, suicidal tendency, post-traumatic stress, sleep problems, social phobia, antisocial tendency, emergency-dealing ability, stress tolerance ability, calm ability, adapting ability, physical control ability, cooperation ability, intelligence, loyalty, courage, confidence, and frustration.

**Table 1:** Baseline characteristics of recruited participants.

**Variables**	**Categories**	**Normal (*n =* 1,268)**	**Mental disorders (*n =* 162)**	***P*** **-value**
Serving place	Gansu	498 (39.27)	41 (25.31)	< 0.001
	Sichuan	607 (47.87)	66 (40.74)	
Chongqing	163 (12.85)	55 (33.95)
Gender	Man	1,213 (95.66)	146 (90.12)	0.002
	Woman	55 (4.34)	16 (9.88)	
Age (years)	< 18.0	105 (8.28)	11 (6.79)	1.000
	18.0–25.0	940 (74.13)	113 (69.75)	
	26.0–30.0	142 (11.20)	20 (12.35)	
	31.0–40.0	71 (5.60)	13 (8.02)	
	41.0–50.0	9 (0.71)	5 (3.09)	
	51.0–60.0	1 (0.08)	0 (0.00)	
Duration of military service (years)^*^	-	2.0 (1.0, 5.0)	3.0 (1.0, 6.0)	0.001
Official rank	Officer	88 (6.94)	13 (8.02)	< 0.001
	Professionals	43 (3.39)	17 (10.49)	
	Civil personnel	0 (0.00)	0 (0.00)	
	Sergeancy	1,101 (86.83)	107 (66.05)	
Soldiers	36 (2.84)	25 (15.43)
Psychosis^*^	-	7.18 (7.18, 7.18)	8.18 (7.18, 9.18)	< 0.001
Depression^*^	-	9.00 (9.00, 10.00)	14.50 (12.00, 18.00)	< 0.001
Suicidal tendency^*^	-	8.05 (8.05, 8.05)	8.05 (8.05, 9.05)	< 0.001
Post-traumatic stress^*^	-	8.00 (8.00, 8.00)	9.00 (8.00, 12.00)	< 0.001
Sleep problems^*^	-	9.50 (9.00, 11.00)	17.00 (12.00, 21.00)	< 0.001
Social phobia^*^	-	10.00 (10.00, 11.00)	15.00 (11.00, 19.00)	< 0.001
Antisocial tendency^*^	-	7.00 (7.00, 7.00)	8.00 (7.00, 11.00)	< 0.001
Emergency-dealing ability^*^	-	16.00 (11.00, 22.00)	22.00 (17.00, 29.00)	< 0.001
Stress tolerance ability^*^	-	18.00 (11.00, 22.00)	24.00 (19.00, 30.00)	< 0.001
Calm ability^*^	-	14.00 (9.00, 17.00)	20.00 (15.00, 25.00)	< 0.001
Adapting ability^*^	-	8.00 (5.00, 10.00)	11.50 (8.00, 16.00)	< 0.001
Physical control ability^*^	-	13.00 (8.50, 16.00)	19.50 (13.00, 25.00)	< 0.001
Cooperation ability^*^	-	8.00 (6.00, 10.00)	11.00 (8.00, 13.00)	< 0.001
Intelligence^*^	-	28.00 (19.00, 31.00)	30.92 (27.00, 38.00)	< 0.001
Loyalty^*^	-	15.00 (12.00, 22.00)	18.00 (16.00, 24.00)	< 0.001
Courage^*^	-	15.00 (9.00, 18.00)	18.00 (14.00, 23.00)	< 0.001
Confidence^*^	-	21.00 (15.00, 28.00)	28.00 (22.00, 34.00)	< 0.001
Frustration^*^	-	18.00 (12.00, 22.00)	24.00 (19.00, 31.00)	< 0.001

* Medians (quartile).

### Mental disorder status

The mental disorders in each item from SCL-90 according to service location are summarized in Table 2. The prevalence of mental disorders is listed as follows: as indicated by percentages in items for somatization (4.06%), obsessive-compulsive (7.06%), interpersonal sensitivity (4.55%), depression (3.43%), anxiety (3.50%), hostility (3.78%), phobic anxiety (1.54%), paranoid ideation (2.94%), psychoticism (1.47%), and additional items (4.76%).

**Table 2:** Frequencies and proportions of mental disorders in Chinese army soldiers assessed using the Symptom Checklist-90 according to serving place.

**Items**	**Group**	**Gansu (*n =* 539)**	**Sichuan (*n =* 673)**	**Chongqing (*n =* 218)**
Somatization	Normal	529 (98.14)	636 (94.50)	207 (94.95)
	Mental disorders	10 (1.86)	37 (5.50)	11 (5.05)
Obsessive compulsive	Normal	517 (95.92)	632 (93.91)	180 (82.57)
	Mental disorders	22 (4.08)	41 (6.09)	38 (17.43)
Interpersonal sensitivity	Normal	522 (96.85)	646 (95.99)	197 (90.37)
	Mental disorders	17 (3.15)	27 (4.01)	21 (9.63)
Depression	Normal	530 (98.33)	648 (96.29)	203 (93.12)
	Mental disorders	9 (1.67)	25 (3.71)	15 (6.88)
Anxiety	Normal	528 (97.96)	649 (96.43)	203 (93.12)
	Mental disorders	11 (2.04)	24 (3.57)	15 (6.88)
Hostility	Normal	535 (99.26)	640 (95.10)	201 (92.20)
	Mental disorders	4 (0.74)	33 (4.90)	17 (7.80)
Phobic anxiety	Normal	533 (98.89)	662 (98.37)	213 (97.71)
	Mental disorders	6 (1.11)	11 (1.63)	5 (2.29)
Paranoid ideation	Normal	533 (98.89)	652 (96.88)	203 (93.12)
	Mental disorders	6 (1.11)	21 (3.12)	15 (6.88)
Psychoticism	Normal	536 (99.44)	666 (98.96)	207 (94.95)
	Mental disorders	3 (0.56)	7 (1.04)	11 (5.05)
Additional items	Normal	520 (96.47)	643 (95.54)	199 (91.28)
	Mental disorders	19 (3.53)	30 (4.46)	19 (8.72)

### Risk factors

Table 3 shows the risk factors for the mental disorders analyzed by univariate logistic regression. We noted that the serving location, gender, age, duration of military service, psychosis, depression, suicidal tendency, post-traumatic stress, sleep problems, social phobia, antisocial tendency, emergency-dealing ability, stress tolerance ability, calm ability, adapting ability, physical control ability, cooperation ability, intelligence, loyalty, courage, confidence, and frustration are significantly associated with the risk of mental disorders. However, the official rank was not associated with the risk of mental disorders. After the multivariate logistic regression was performed, we noted that serving place (Sichuan vs. Gansu: OR, 1.846, 95% CI: 1.028–3.315, *P* = 0.038; Chongqing vs. Gansu: OR, 3.129, 95% CI, 1.669–5.869, *P* = 0.003), increased psychosis (OR, 1.491, 95% CI, 1.152–1.928, *P* = 0.002), depression (OR, 1.482, 95% CI, 1.349–1.629, *P* < 0.001), sleep problems (OR, 1.235, 95% CI, 1.162–1.311, *P* < 0.001), and frustration scores (OR, 1.050, 95% CI, 1.015–1.087, *P* = 0.005) are associated with an increased risk of mental disorders (Table 4).

**Table 3:** Risk factors for mental disorders in Chinese army soldiers assessed using the Symptom Checklist-90 by univariate logistic regression.

**Variable**	**β**	**SE**	**Statistic**	***P*** **-value**	**OR**	**95%LCI**	**95%UCI**
Serving place	0.718	0.121	35.290	< .001	2.050	1.618	2.597
Gender	0.883	0.297	8.817	0.003	2.417	1.350	4.328
Age (years)	0.260	0.107	5.867	0.015	1.297	1.051	1.600
Duration of military service (years)	0.048	0.016	9.068	0.003	1.049	1.017	1.083
Official rank	−0.059	0.090	0.434	0.510	0.942	0.790	1.124
Psychosis	1.245	0.111	126.155	< .001	3.473	2.795	4.316
Depression	0.614	0.041	225.811	< .001	1.848	1.706	2.002
Suicidal tendency	0.781	0.104	55.974	< .001	2.183	1.779	2.678
Post-traumatic stress	0.547	0.050	119.510	< .001	1.728	1.567	1.906
Sleep problems	0.374	0.026	203.860	< .001	1.454	1.381	1.531
Social phobia	0.465	0.034	190.728	< .001	1.592	1.490	1.701
Antisocial tendency	0.733	0.065	129.276	< .001	2.082	1.835	2.363
Emergency-dealing ability	0.098	0.010	94.674	< .001	1.102	1.081	1.124
Stress tolerance ability	0.108	0.011	96.871	< .001	1.115	1.091	1.139
Calm ability	0.140	0.013	112.242	< .001	1.150	1.121	1.180
Adapting ability	0.199	0.019	108.806	< .001	1.220	1.175	1.266
Physical control ability	0.164	0.015	126.788	< .001	1.178	1.145	1.212
Cooperation ability	0.166	0.020	68.430	< .001	1.180	1.135	1.227
Intelligence	0.062	0.0081	58.102	< .001	1.064	1.047	1.081
Loyalty	0.067	0.011	37.603	< .001	1.069	1.046	1.092
Courage	0.125	0.014	77.933	< .001	1.134	1.102	1.166
Confidence	0.079	0.0093	73.071	< .001	1.082	1.063	1.102
Frustration	0.117	0.012	104.306	< .001	1.124	1.099	1.150

**Table 4:** Risk factors for mental disorders in Chinese army soldiers assessed using the Symptom Checklist-90 by multivariate logistic regression.

**Variable**	**β**	**SE**	**Statistic**	***P*** **-value**	**OR**	**95%LCI**	**95%UCI**
Serving place	0.528	0.171	2.027	0.038	1.846	1.028	3.315
0.556	0.184	9.167	0.003	3.129	1.669	5.869
Psychosis	0.399	0.131	9.245	0.002	1.491	1.152	1.928
Depression	0.394	0.048	66.640	< 0.001	1.482	1.349	1.629
Sleep problems	0.211	0.031	46.877	< 0.001	1.235	1.162	1.311
Frustration	0.049	0.017	7.960	0.005	1.050	1.015	1.087

### Prediction model

**Figure 1:**
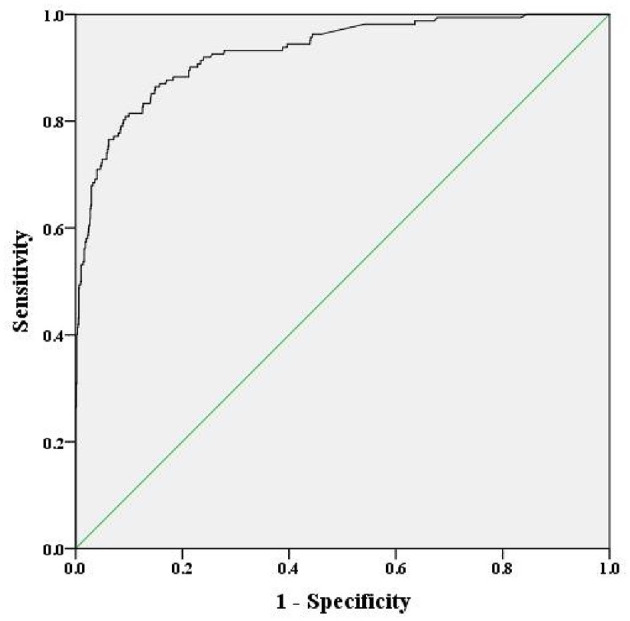
Receiver operating curve of the risk for mental disorders, including the five-component risk factor model (area under the curve = 0.930).

We combined service location, psychosis, depression, sleep problems, and frustration to construct the prediction model, and the result is shown in Figure 1. The predictive value of this model was high, and the AUC was 0.930 (95% CI: 0.907–0.952) for predicting mental disorders in Chinese soldiers.

## Discussion

The prevalence of mental disorders in Chinese soldiers was relatively high. Therefore, researchers should assess potential risk factors and develop a prediction model for their early management to improve the progression and prognosis for mental disorders. We identified 1,430 Chinese soldiers from three service locations. The prevalence of mental disorders was 11.33%. In addition, the prevalence of obsessive-compulsive, interpersonal sensitivity, depression, anxiety, hostility, phobic anxiety, paranoid ideation, psychoticism, and additional items was highest in Chongqing, while somatization was highest in Sichuan. The risk factors for mental disorders in Chinese soldiers included service location, psychosis, depression, sleep problems, and frustration. Combining these factors, the predictive value of our model was high, and the AUC was 0.930.

The univariate analysis indicated the risk of mental disorders could be affected by serving location, gender, age, duration of military service, psychosis, depression, suicidal tendency, post-traumatic stress, sleep problems, social phobia, antisocial tendency, emergency-dealing ability, stress tolerance ability, calm ability, adapting ability, physical control ability, cooperation ability, intelligence, loyalty, courage, confidence, and frustration, while after adjusting the confounding factors, we noted that service location, psychosis, depression, sleep problems, and frustration are associated with increased mental disorders. Several reasons could explain these results: (1) Military service duration in Chongqing was longer than in Sichuan and Gansu, and more than 50% of the Chongqing portion of the sample included soldiers aged 25 years or more, while this proportion in Sichuan and Gansu was < 30%; thus, the soldiers' age might affect the risk of mental disorders (27). Moreover, the proportion of women in Chongqing (22.94%) was higher than in Sichuan (1.63%) and Gansu (1.86%), meaning the prevalence of mental disorders could be affected by gender (28); (2) the hallucinations and delusions of psychosis could cause an additional risk of mental disorders (29); (3) sleep problems could be induced by an underlying illness or history of mental illness, which might play an important role in promoting panic, anxiety, and depression (30); and (4) frustration is significantly associated with emotional difficulties, which could contribute to the progression of mental disorders (31).

Our study constructed a prediction model for mental disorders in Chinese soldiers. Several similar studies have already been performed on the general population (32, 33). For example, Karasch et al. identified 5,764 cases admitted to inpatient care at the four psychiatric hospitals and found that specific psychiatric diagnoses and suicidal tendencies are major risk factors for involuntary psychiatric hospitalization (32). In addition, Puntis et al. tested an individualized, clinically based trans-diagnostic model for detecting individuals at risk of psychosis and found the prognostic performance was adequate (33). The prediction model in our study showed greater predictive power than the previous models, which indicates the items from three questionnaires could be applied to monitor mental disorders in Chinese soldiers.

The results of this study provide more information for army administrators. The risk factors for mental disorders in Chinese soldiers were identified. Moreover, the combined prediction model was constructed, which could screen soldiers at high risk for mental disorders. Considering the results of this study, early psychological counseling and intervention should be applied for soldiers at high risk to prevent the progression of mental disorders.

We should acknowledge several shortcomings of this study. First, this study was cross-sectional and cannot confirm a causal relationship for predictors. Second, the analysis was not divided into a training and validation set, and an external cohort study should be performed to validate this model. Finally, the SCL-90 has inherent limitations, including cultural adaptation. Finally, all questionnaires hold the risk of “fake good” bias, where the respondent provides answers they think the researcher would like to read or hear, and memory failure.

## Conclusion

The study findings reported the prevalence of mental disorders in Chinese soldiers. The identified risk factors included service location, psychosis, depression, sleep problems, and frustration. The prediction model based on these factors provides high predictive value for mental disorders in Chinese soldiers. However, the robustness of the prediction model should be validated in further large-scale prospective studies.

## Strengths and limitations of this study

(1) The prevalence of mental disorders in Chinese soldiers was reported.
(2) Identified risk factors included service location, psychosis, depression, sleep problems, and frustration.
(3) The prediction model provides high predictive value for mental disorders in Chinese soldiers.
(4) This study was cross-sectional and cannot confirm a causal relationship between predictors.
(5) The analysis was not divided into a training and validation set, and the model requires further validation.

## Data availability statement

The raw data supporting the conclusions of this article will be made available by the authors, without undue reservation.

## Ethics statement

The studies involving human participants were reviewed and approved by the Third Military Medical University. The patients/participants provided their written informed consent to participate in this study.

## Author contributions

MZ: study conception, design, processing data, analysis, drafting, and manuscript revision. ZF, YH, and QT: study conception, design, and manuscript revision. NW: data acquisition, processing data, and psychometric test descriptions. HZ: data acquisition and manuscript revision. All authors contributed to the article and approved the submitted version.
