# Supplementary material for: Risk factors and prediction model for mental health in Chinese soldiers
Source: Front Psychiatry. 2023 May 5;14:1125411. doi: 10.3389/fpsyt.2023.1125411 (PMC10196266; doi:10.3389/fpsyt.2023.1125411)
Supplement: Supplementary file 1 [file Table_1.DOCX]

1. Military Mental Health Status Questionnaire

Instruction: Please tick "√" on the option that best matches your feelings in the last week. From none (no symptom), mild (have this symptom, but not frequent and serious), moderate (have this symptom, but moderate severity), severe (often have this symptom, but serious severity), serious (very serious frequency and intensity for symptoms).

| **Items** | **None** | **Mild** | **Moderate** | **Severe** | **Serious** |
| --- | --- | --- | --- | --- | --- |
| 1. I thought my ability to speak was controlled by others, that I was just a talking machine. |  |  |  |  |  |
| 1. I am often deliberately pretentious or strangely imitative of the normal behaviour of others. |  |  |  |  |  |
| 1. I often get weird feelings, like the feeling that a part of my body is dead or unreal. |  |  |  |  |  |
| 1. I often talk to people that no one else can see. |  |  |  |  |  |
| 1. More than once, when awake, I hear sounds that others cannot hear, such as noises, music, or conversations. |  |  |  |  |  |
| 1. I don't think I can control my thoughts and unconsciously jump from one topic to another when I speak. |  |  |  |  |  |
| 1. I have often felt that I must speak uncontrollably fast, so people often say that it is difficult to understand what I am saying. |  |  |  |  |  |
| 1. I have no enthusiasm or interest in doing things |  |  |  |  |  |
| 1. I feel low, depressed or hopeless. |  |  |  |  |  |
| 1. I feel very tired or lack energy. |  |  |  |  |  |
| 1. I lose interest in important activities that I used to enjoy, such as work, hobbies, sports or social activities. |  |  |  |  |  |
| 1. I have trouble focusing on one thing or thing, and my concentration drops, such as when reading a newspaper or watching TV. |  |  |  |  |  |
| 1. I consider myself a failure and worthless. |  |  |  |  |  |
| 1. I was no longer truly satisfied with anything. |  |  |  |  |  |
| 1. I felt there was nothing to look forward to. |  |  |  |  |  |
| 1. Even if something exciting happens, I don't expect much. |  |  |  |  |  |
| 1. I seriously considered taking my own life. |  |  |  |  |  |
| 1. I don't think suicide causes anyone pain. |  |  |  |  |  |
| 1. I will intentionally injure myself or allow my body to be "accidentally" damaged. |  |  |  |  |  |
| 1. I had made specific plans to kill myself. |  |  |  |  |  |
| 1. When I make plans to kill myself, I am relaxed or happy. |  |  |  |  |  |
| 1. In the past period of time, there have been acts of intentional self-harm. |  |  |  |  |  |
| 1. I think killing yourself or hurting yourself is the best way to solve the problem. |  |  |  |  |  |
| 1. When life ends at that moment, all my troubles are gone. |  |  |  |  |  |
| 1. I would suddenly feel like I was back at a traumatic military training or mission, with all the fear and fear I felt when it happened. |  |  |  |  |  |
| 1. I get upset when I see or hear about someone or something related to a traumatic military experience |  |  |  |  |  |
| 1. I get nervous, scared, upset, etc. when I suddenly think of someone involved in a traumatic military experience or something similar. |  |  |  |  |  |
| 1. Try to avoid training or missions similar to a traumatic military experience. |  |  |  |  |  |
| 1. Try to avoid thinking or talking about a past military experience that involved death or major injury. |  |  |  |  |  |
| 1. Try to avoid activities or situations that remind me of past traumatic military training or missions. |  |  |  |  |  |
| 1. After a traumatic military event, I am easily angry, impulsive, or irritable. |  |  |  |  |  |
| 1. I felt nervous or extremely frightened, constantly worried that the same bloodshed or even death would happen during training or missions. |  |  |  |  |  |
| 1. I feel like I'm not getting enough sleep. |  |  |  |  |  |
| 1. I think my sleep is very bad, even seriously affect the next day's work and life. |  |  |  |  |  |
| 1. Even if you sleep for more than eight hours, you still feel sleepy when you wake up during the day. |  |  |  |  |  |
| 1. I have trouble falling asleep in 30 minutes |  |  |  |  |  |
| 1. I was troubled by my difficulty falling asleep which affected my sleep quality. |  |  |  |  |  |
| 1. When I went to bed that night, I was more and more awake for fear of insomnia. |  |  |  |  |  |
| 1. I wake up early in the morning, often earlier than expected. |  |  |  |  |  |
| 1. I often have strange or horrible nightmares. |  |  |  |  |  |
| 1. During the night, I was easily awakened by even a small noise and had difficulty falling back asleep. |  |  |  |  |  |
| 1. At social events, I suddenly feel fear, fear. |  |  |  |  |  |
| 1. When I think about interacting or interacting with strangers, I feel anxious and nervous. |  |  |  |  |  |
| 1. When I'm about to speak or perform in public, I feel my heart racing, I sweat, I have trouble breathing, I feel weak or I shake. |  |  |  |  |  |
| 1. When interacting with people, I spend a lot of time thinking about what I should say or do. |  |  |  |  |  |
| 1. Even when I go into a social place, I try to avoid talking or interacting with people. |  |  |  |  |  |
| 1. I try to be alone or avoid social activities. |  |  |  |  |  |
| 1. When something unexpected happens to me in a social situation, I have thoughts of being humiliated, embarrassed, laughed at, or offended. |  |  |  |  |  |
| 1. I try to find something I can do alone to distract myself from thinking about social situations. |  |  |  |  |  |
| 1. I feel uncomfortable communicating with people. |  |  |  |  |  |
| 1. It is only under the influence of alcohol, drugs, etc., that I dare to engage in social activities and interact with strangers. |  |  |  |  |  |
| 1. I often deceive others for personal gain or interest. |  |  |  |  |  |
| 1. No matter what I do, I'm always rude or unfriendly. |  |  |  |  |  |
| 1. I get angry easily and even the smallest incident sets me off. |  |  |  |  |  |
| 1. I have a tendency to attack or beat someone when they do something that annoys me. |  |  |  |  |  |
| 1. I tend to lose control of myself and become impulsive. |  |  |  |  |  |
| 1. I don't care if I hurt anyone. |  |  |  |  |  |
| 1. I'm not responsible for anything. |  |  |  |  |  |

1. Military Mental Health Ability Questionnaire

Guide: Please read each question carefully, according to your own situation, choose one of the "never, rarely, sometimes, often, always", and tick "√" on the corresponding position.

| **Items** | **Never** | **Rarely** | **Sometimes** | **Often** | **Always** |
| --- | --- | --- | --- | --- | --- |
| 1.I think it's more efficient to work together than to work alone |  |  |  |  |  |
| 2.Even if the rules and regulations do not agree with some of my ideas, I am quick to accept them and follow them |  |  |  |  |  |
| 3.I believe that the individual has an obligation to make sacrifices when his own interests conflict with those of the group |  |  |  |  |  |
| 4.I can handle my negative emotions |  |  |  |  |  |
| 5.I am an even-tempered person |  |  |  |  |  |
| 6.I think it's an honorable thing to make a contribution to the collective |  |  |  |  |  |
| 7.When the job changes, I can move into the new role more quickly |  |  |  |  |  |
| 8.I will adjust my habits according to the changes in my life situation |  |  |  |  |  |
| 9.I adjust my life goals according to the changing reality of the situation |  |  |  |  |  |
| 10.I can adapt to intense military training |  |  |  |  |  |
| 11.I can find the positive in the bad things |  |  |  |  |  |
| 12.I think my words and actions will affect the development of the group |  |  |  |  |  |
| 13.I feel that my own success or failure depends on my fellow soldiers |  |  |  |  |  |
| 14.In the face of a crisis, I often feel my potential has been unleashed and I perform better than usual |  |  |  |  |  |
| 15.When I feel fear, I try to bring my attention back to the task at hand |  |  |  |  |  |
| 16.When I face a difficulty, I can recall a time when I faced similar difficulties and overcame them |  |  |  |  |  |
| 17.I believe that personal goals should be as consistent as possible with team goals |  |  |  |  |  |
| 18.When I feel extremely tired, I can still cheer myself up |  |  |  |  |  |
| 19.In the face of sudden changes in the work, I dare to break the original plan and adapt to the situation |  |  |  |  |  |
| 20.In the face of great pressure, I can calm down and do the job at hand |  |  |  |  |  |
| 21.Under pressure, I can focus and think clearly |  |  |  |  |  |
| 22.I can accept sudden changes very quickly |  |  |  |  |  |
| 23.I can remain calm under great pressure |  |  |  |  |  |
| 24.I was able to overcome my inner loneliness when I was on dangerous missions alone |  |  |  |  |  |
| 25.I am the one who dares to take action in the face of things that others find difficult |  |  |  |  |  |
| 26.I can make unusual or difficult decisions |  |  |  |  |  |
| 27.Given a life-threatening assignment, I can jump into action |  |  |  |  |  |
| 28.I can deal with unexpected events calmly |  |  |  |  |  |
| 29.When I'm a prisoner, I try to calm myself down and watch what happens |  |  |  |  |  |
| 30.Being sent suddenly to a place where living conditions are difficult, I can adapt quickly |  |  |  |  |  |
| 31.I was quick to talk myself into accepting the separation of troops from family or close comrades |  |  |  |  |  |
| 32.I was able to recover quickly from an intense training session and jump into training the next day |  |  |  |  |  |
| 33.When I go to extreme environments (such as high altitude, desert, island, etc.), I can quickly adapt to the local life |  |  |  |  |  |
| 34.Back in the army after leave, I was able to get used to the new pace of life quickly |  |  |  |  |  |
| 35.I'm a better runner than most of my fellow soldiers |  |  |  |  |  |
| 36.I'm better coordinated than most of my fellow soldiers |  |  |  |  |  |
| 37.My body has more strength than most of my comrades |  |  |  |  |  |
| 38.I was able to learn complex body movements (such as tactical training moves) faster than most of my comrades |  |  |  |  |  |
| 39.I feel confident when I take part in sports |  |  |  |  |  |
| 40.I am confident that I can maintain my good physical condition |  |  |  |  |  |
| 41.I feel good about my physical health |  |  |  |  |  |
| 42.When I communicate with people, I make sure they understand what I'm trying to say |  |  |  |  |  |
| 43.I think the difficulty is temporary and I will come out of it eventually |  |  |  |  |  |
| 44.I'm a good listener when I'm talking to people |  |  |  |  |  |
| 45.In the face of great pressure, I was still able to complete tasks methodically |  |  |  |  |  |
| 46.Before I do something, I consider whether it will affect the interests of my fellow soldiers |  |  |  |  |  |

1. Mental Quality Questionnaire for Army-Men

Guide: Please read each question carefully, according to your own situation, and then according to the sentence is consistent with your actual situation, underline the number you think is the most consistent. The numbers represent the following meanings: 1 - very inconsistent, 2 - relatively inconsistent, 3 - uncertain, 4 - fairly consistent, 5 - very consistent. You can only choose one answer to each question. Try not to select "not sure" unless you think the other four options are really not what you want;

There is no time limit for this test, but don't think too much about the question, just answer it truthfully; There are no right or wrong answers, so you don't have to worry.

Please be sure to answer every question and leave nothing out.

|  | **Items** | **Grading** | | | | |
| --- | --- | --- | --- | --- | --- | --- |
| 1 | I like to observe or ask questions. | 1 | 2 | 3 | 4 | 5 |
| 2 | I can always see where things are going. | 1 | 2 | 3 | 4 | 5 |
| 3 | I like to analyze problems from different angles. | 1 | 2 | 3 | 4 | 5 |
| 4 | I can always make the right judgment based on the situation. | 1 | 2 | 3 | 4 | 5 |
| 5 | I am used to judging people and things from many aspects. | 1 | 2 | 3 | 4 | 5 |
| 6 | Always gather a lot of information before making a decision. | 1 | 2 | 3 | 4 | 5 |
| 7 | Before I make a decision, I will carefully consider the feasibility of various options. | 1 | 2 | 3 | 4 | 5 |
| 8 | When dealing with a problem, I am used to proposing multiple solutions and then choosing the best one. | 1 | 2 | 3 | 4 | 5 |
| 9 | I will take the initiative to check the merits and demerits of the decisions made. | 1 | 2 | 3 | 4 | 5 |
| 10 | When there is a problem in the implementation of the decision, I will timely correct and adjust. | 1 | 2 | 3 | 4 | 5 |
| 11 | I do things by analogy. | 1 | 2 | 3 | 4 | 5 |
| 12 | I am good at using what I have learned to solve practical problems in life. | 1 | 2 | 3 | 4 | 5 |
| 13 | I like to find multiple solutions to problems. | 1 | 2 | 3 | 4 | 5 |
| 14 | I think I can handle problems better than my peers. | 1 | 2 | 3 | 4 | 5 |
| 15 | I always take problems in my stride. | 1 | 2 | 3 | 4 | 5 |
| 16 | I am proud to be Chinese. | 1 | 2 | 3 | 4 | 5 |
| 17 | I pay close attention to the country's development situation, current affairs and policies and some social problems. | 1 | 2 | 3 | 4 | 5 |
| 18 | I am willing to contribute to society. | 1 | 2 | 3 | 4 | 5 |
| 19 | Whenever the national flag is raised and the national anthem is played, I feel very sacred. | 1 | 2 | 3 | 4 | 5 |
| 20 | I have great respect for those war heroes, revolutionary leaders and patriots. | 1 | 2 | 3 | 4 | 5 |
| 21 | I always like to give in to others. | 1 | 2 | 3 | 4 | 5 |
| 22 | In my life, I often help others. | 1 | 2 | 3 | 4 | 5 |
| 23 | I volunteered for the army. | 1 | 2 | 3 | 4 | 5 |
| 24 | I'm willing to go the extra mile to help the group get the job done. | 1 | 2 | 3 | 4 | 5 |
| 25 | If given the chance, I will volunteer to blood. | 1 | 2 | 3 | 4 | 5 |
| 26 | I am a very responsible person. | 1 | 2 | 3 | 4 | 5 |
| 27 | When colleagues and friends ask me for advice, I always prepare carefully and answer earnestly. | 1 | 2 | 3 | 4 | 5 |
| 28 | I always finish my tasks strictly according to schedule. | 1 | 2 | 3 | 4 | 5 |
| 29 | I think everyone has a duty to prosper the country. | 1 | 2 | 3 | 4 | 5 |
| 30 | When I make a mistake in my work, I will take the responsibility bravely. | 1 | 2 | 3 | 4 | 5 |
| 31 | I am a man of quick decisions. | 1 | 2 | 3 | 4 | 5 |
| 32 | When I meet the bad guys, I can figure out what to do, depending on the situation. | 1 | 2 | 3 | 4 | 5 |
| 33 | I always solve problems at work with a clean SLATE. | 1 | 2 | 3 | 4 | 5 |
| 34 | In dealing with life, I like to "cut through the clutter". | 1 | 2 | 3 | 4 | 5 |
| 35 | Even if the situation is chaotic, I can distinguish right from wrong and act in time. | 1 | 2 | 3 | 4 | 5 |
| 36 | I like to do one thing well before I do another. | 1 | 2 | 3 | 4 | 5 |
| 37 | Even if there is no supervision or inspection, I will finish the task on time. | 1 | 2 | 3 | 4 | 5 |
| 38 | Even in the face of difficulties, I will try to finish the plan. | 1 | 2 | 3 | 4 | 5 |
| 39 | I always stick to the plan and don't give up easily. | 1 | 2 | 3 | 4 | 5 |
| 40 | I've always been able to hold my own when the going gets tough. | 1 | 2 | 3 | 4 | 5 |
| 41 | I like a challenging job. | 1 | 2 | 3 | 4 | 5 |
| 42 | In the face of great obstacles, I will choose to fight. | 1 | 2 | 3 | 4 | 5 |
| 43 | In a 5k cross country, even if I fall behind, I will keep running the whole distance. | 1 | 2 | 3 | 4 | 5 |
| 44 | I always keep my spirits up in the face of adversity. | 1 | 2 | 3 | 4 | 5 |
| 45 | I'm willing to leave no stone unturned to tackle a problem. | 1 | 2 | 3 | 4 | 5 |
| 46 | My comrades thought I was a man who could keep his cool. | 1 | 2 | 3 | 4 | 5 |
| 47 | I am good at controlling my words and actions. | 1 | 2 | 3 | 4 | 5 |
| 48 | I'm used to making decisions after careful consideration. | 1 | 2 | 3 | 4 | 5 |
| 49 | I can handle things with peace of mind. | 1 | 2 | 3 | 4 | 5 |
| 50 | I am also able to keep calm in the completion of time-pressed tasks. | 1 | 2 | 3 | 4 | 5 |
| 51 | When discussing problems with others, I have my own opinions. | 1 | 2 | 3 | 4 | 5 |
| 52 | I can take charge of my own work. | 1 | 2 | 3 | 4 | 5 |
| 53 | I can also think independently when cooperating with others. | 1 | 2 | 3 | 4 | 5 |
| 54 | When I have a problem, I like to think alone. | 1 | 2 | 3 | 4 | 5 |
| 55 | I believe I can complete challenging tasks independently. | 1 | 2 | 3 | 4 | 5 |
| 56 | Most people who know me like me. | 1 | 2 | 3 | 4 | 5 |
| 57 | My roommates all say that I am an easy-going person. | 1 | 2 | 3 | 4 | 5 |
| 58 | People around me always ask me for advice or help when they have problems. | 1 | 2 | 3 | 4 | 5 |
| 59 | My comrades seldom come to me for advice. | 1 | 2 | 3 | 4 | 5 |
| 60 | I can get along well with comrades with different personalities. | 1 | 2 | 3 | 4 | 5 |
| 61 | I felt like a duck to water in the army. | 1 | 2 | 3 | 4 | 5 |
| 62 | Even in the noisy environment, my study and work can not be affected. | 1 | 2 | 3 | 4 | 5 |
| 63 | In a new environment, I can eat and sleep well even if my diet, work and rest have changed a lot. | 1 | 2 | 3 | 4 | 5 |
| 64 | The rules and regulations of the army are not always reasonable, but I always try to follow them. | 1 | 2 | 3 | 4 | 5 |
| 65 | I can fit into different groups very quickly. | 1 | 2 | 3 | 4 | 5 |
| 66 | In times of adversity, my actions are not controlled by my emotions. | 1 | 2 | 3 | 4 | 5 |
| 67 | Even when things are bad, I can still live a positive life. | 1 | 2 | 3 | 4 | 5 |
| 68 | When goals don't come true, I accept them calmly. | 1 | 2 | 3 | 4 | 5 |
| 69 | I was able to work calmly after being criticized by the leader. | 1 | 2 | 3 | 4 | 5 |
| 70 | I don't feel unfair if I don't get an A grade even though I've done well. | 1 | 2 | 3 | 4 | 5 |
| 71 | When life or business fails, I can objectively analyze the reasons and keep a normal attitude. | 1 | 2 | 3 | 4 | 5 |
| 72 | When I am frustrated, I can find many happy and interesting things. | 1 | 2 | 3 | 4 | 5 |
| 73 | I agree with the saying "it's a blessing in disguise". | 1 | 2 | 3 | 4 | 5 |
| 74 | I think that if I do my best, I can have a clear conscience. | 1 | 2 | 3 | 4 | 5 |
| 75 | When I encounter obstacles in my work, I will turn to others for help. | 1 | 2 | 3 | 4 | 5 |
